# Neurolymphomatosis mimicking neurosarcoidosis: a case report

**DOI:** 10.1186/1752-1947-4-5

**Published:** 2010-01-12

**Authors:** Ernestina Santos, Neil J Scolding

**Affiliations:** 1 Neurology Department, Hospital Geral Santo, António Porto, Portugal; 2 University of Bristol Institute of Clinical Neurosciences, Neurology Department, Frenchay Hospital, Bristol BS16 1LE, UK

## Abstract

**Introduction:**

Both neurosarcoidosis and central nervous system lymphoma can be very difficult to diagnose. We describe the case of a patient in whom neurosarcoidosis was strongly suspected, but who was eventually found to have lymphoma. We believe the case to be of interest and practical value to neurologists, oncologists and internists with an interest in inflammatory diseases.

**Case presentation:**

A diagnosis of neurosarcoidosis was considered in a 49-year-old Caucasian man on the basis of the following symptoms and indications: a cough, bilateral hilar lymphadenopathy confirmed by thoracic computed tomography, the development of an S1 radiculopathy, cerebrospinal fluid abnormalities (raised protein level), bilateral lung hilar and lachrymal gland uptake on a gallium scan, and erythema nodosum confirmed with skin biopsy. These were followed by the development of multiple cranial neuropathies, including seventh nerve palsy. Exhaustive further investigations yielded no evidence for an alternative diagnosis. Treatments with steroids, cyclophosphamide, intravenous immunoglobulin and finally infliximab were of no benefit. He eventually developed cutaneous nodules, a biopsy of which revealed lymphoma that proved resistant to therapy.

**Conclusion:**

Constant diagnostic vigilance is required in disorders such as neurosarcoidosis.

## Introduction

Systemic inflammatory, autoimmune, infectious or neoplastic disorders frequently involve the central nervous system (CNS). Establishing a diagnosis can be particularly difficult when neurological symptoms are the presenting feature. Biological markers or diagnostic evidence of other organ involvement can be absent, and the perceived hazards, combined with the potential for eliciting only non-diagnostic information, often mitigate against cerebral biopsy.

We present the case of a patient that illustrates such difficulties and we discuss the implications of using aggressive immunosuppressive therapy in patients with suspected inflammatory disease.

## Case presentation

A 49-year-old Caucasian man developed a cough in early 2004. A chest X-ray revealed bilateral hilar lymphadenopathy, confirmed by thoracic computed tomography (CT) scan. He had no other symptoms. A diagnosis of sarcoidosis was considered, but his symptoms were thought insufficient to warrant treatment.

In July 2004, he developed numbness and pain behind the right knee which gradually spread to the lower back, right buttock and posterior thigh. Upon examination he had reduced sensation over the lateral border of the right foot, an absent right ankle tendon reflex and a positive Lasègue's sign at 70°. He also had a dusky discolouration of the skin of the right foot.

He was admitted to our hospital in September 2004 because of progressive worsening of the symptoms. Lumbrosacral spinal magnetic resonance imaging (MRI) showed an increased heterogeneous signal within the S1 nerve root and of the nerve root ganglion on T2 images, thought to be due to oedema, with right piriformis wasting. His cerebrospinal fluid (CSF) contained no white cells, 0.52 g/l protein and 0.36 g/l glucose. Nerve conduction studies and an electromyogram (EMG) revealed abnormalities in the S1 segment, consistent with an S1 radiculopathy. Serum angiotensin converting enzyme (sACE) was persistently normal but an isotope-labelled gallium scan showed increased bilateral lung hilar and lachrymal gland uptake. He developed skin nodules on his right thigh which, when biopsied, were confirmed as erythema nodosum. The diagnosis of sarcoidosis was considered overwhelmingly likely, and in the absence of compression, the involvement of the S1 root was thought most likely due to neurosarcoidosis. He started treatment with prednisone 30 mg/day but his pain persisted; intravenous steroids and then local steroid nerve root injection was tried with temporary benefit. In April 2005, he started methotrexate (up to 12.5 mg per week) because of persistent pain and the need to lower his steroid dose because of his elevated glucose levels.

In November 2005, he developed left peri-orbital and hemicranial headache, followed by diplopia on left gaze. He was found to have a partial left sixth nerve palsy and was re-admitted. MRI showed thickening and gadolinium enhancement in the left cavernous sinus with no parenchymal change. Repeat gallium scanning showed normal lung hilar and lachrymal gland uptake. Serum rheumatoid factor, plasma viscosity, C-reactive protein, urea and electrolytes, liver function, clotting, auto-immune profile, protein electrophoresis, acetylcholine receptor antibodies, anti-neuronal antibodies, anti-thyroid antibodies, creatine kinase and ACE were all normal. He was treated with a three-day course of intravenous methylprednisone and experienced significant improvement.

The following month, he developed headache and further diplopia and he was found to have a painful pupil-sparing left third nerve palsy. His CSF was again entirely normal, including negative oligoclonal band assay. Brain and orbit MRI scanning were normal. EMG and nerve conduction studies suggested improvement of S1 radiculopathy. He also complained of left facial pain with tearing of the left eye. There was a patchy decrease in sensation on the left side of the face and scalp. Corneal reflex was diminished. Blink reflex and facial nerve conduction studies showed an afferent defect on the left suggesting a left trigeminal ophthalmic division neuropathy. His prednisone was increased to 60 mg/day, and cyclophosphamide was started instead of methotrexate. However, after reducing his steroids to 40 mg/day, severe facial pain recurred.

In March 2006, he developed numbness and tingling on the left side of his face, and was found to have a left maxillary ophthalmic division Vth neuropathy. A month later he developed a lower motor neuron left seventh nerve palsy and numbness in the right shoulder and in the left thorax. His CSF was again normal and/or negative including cytological study, acid-fast bacilli staining, and fungal and *Mycobacterium tuberculosis *cultures and sACE was also still normal. His blood count showed mild lymphopenia and macrocytosis. Lactate dehydrogenase was normal. Borrelia, syphilis, cytomegalovirus, HIV and human T-lymphotropic virus Type 1 serology were negative. An ophthalmological examination was normal with no signs of granulomata.

**Figure 1:**
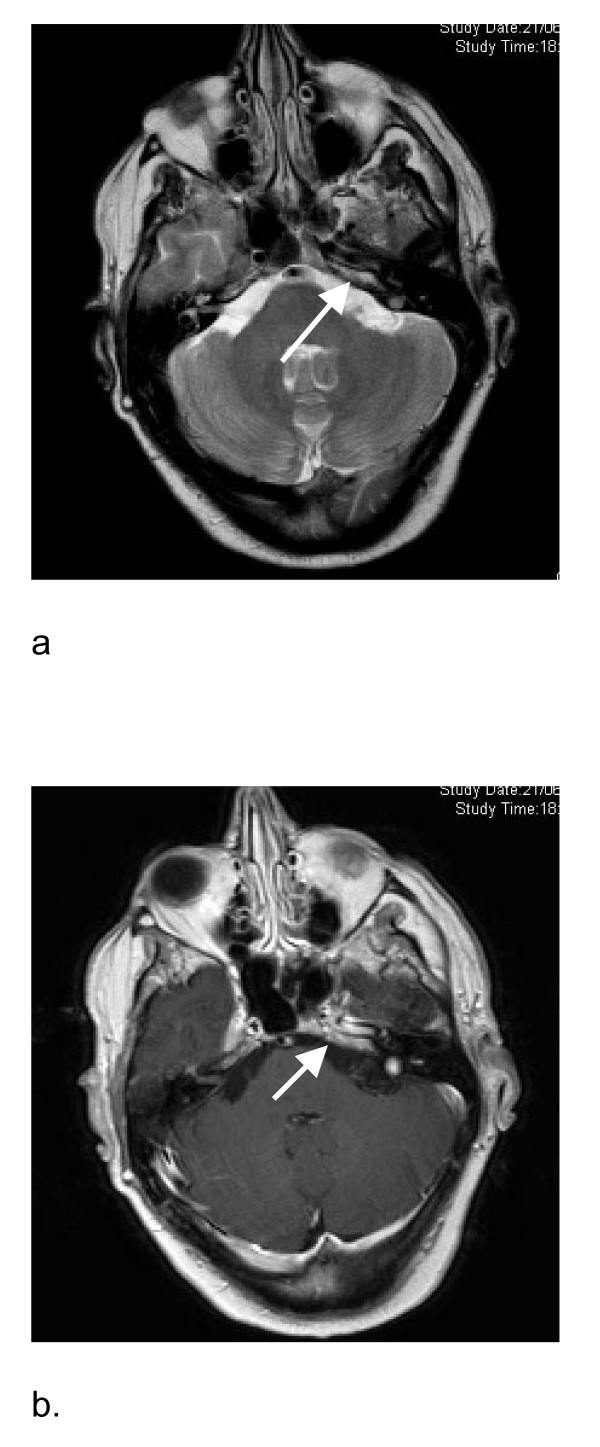
**Brain magnetic resonance images**. **(a) **Brain magnetic resonance image scanning T2 weighted image showing high signal of the fifth, sixth, seventh and eighth cranial nerves (arrow). **(b) **Brain magnetic resonance image scanning T1 after gadolinium showing enhancement of the fifth, sixth, seventh and eighth cranial nerves (arrow).

At this stage, new neurological symptoms developed while the patient was on treatment with steroids and cyclophosphamide. Because no diagnosis emerged other than sarcoidosis, alternative immunosuppressive therapy was administered. Intravenous immunoglobulins, however, had no impact. Infliximab was added in June 2006. Despite this, the patient's right shoulder became weak, his headache persisted and he also developed unsteadiness of gait and significant weight loss. He now had bilateral seventh nerve palsies with weakness of the left palate, right serratus anterior, right triceps, left triceps and left finger abductors. All the upper limb deep tendon jerks were absent. A left vocal cord palsy was noted. EMG and nerve conduction studies showed abnormalities compatible with a pre-ganglionic lesion at C5 and C6 level (right), but no generalised neuropathy. Repeat brain MRI scanning showed enhancement of the fifth, sixth, seventh and eighth cranial nerves (Figure 1). Spine MRI scanning was normal. MRI scanning of the upper brachial plexus was normal. A CT scan of the pelvis and abdomen were normal. A whole body fluorodeoxyglucose-positron emission tomography (FDG-PET) scan was normal. Urine thallium screening and lead level were negative. Upper gastrointestinal endoscopy was normal.

The patient then developed three subcutaneous nodules, one in the left outer inferior breast quadrant, without lymphadenopathy. Biopsy of the nodules showed lymphomatous change, and the final diagnosis was diffuse large B-cell lymphoma involving cranial and peripheral nerves (neurolymphomatosis). Bone marrow was normal.

He was transferred to the Oncology department and treated with chemotherapy including methotrexate. After chemotherapy he received total body irradiation and a bone marrow stem cell transplant. There was no significant improvement in his neurological condition.

## Discussion

Sarcoidosis is an inflammatory systemic disorder of unknown cause. Neurosarcoidosis is a serious complication found in 5 to 15% of patients with sarcoidosis and often has a poor prognosis; treatment is recommended in the early period of disease. It most commonly causes cranial neuropathies, but any part of nervous system can be involved [1]. Peripheral nerve involvement is less common and usually occurs in 15 to 18% of patients [2].

When neurological problems develop in patients with biopsy-proven systemic sarcoidosis, the diagnosis is usually straightforward. However, without biopsy evidence of sarcoidosis in other sites, neurological sarcoidosis may be difficult to diagnose and other disorders difficult to exclude, particularly infection and neoplasia. The importance of histological confirmation before starting treatment cannot be exaggerated [3], and our case helps illustrate this. Tissue-based proof of sarcoidosis was, despite our best efforts, never acquired. Had the patient undergone a transbronchial biopsy at the onset of his illness (when he had bilateral hilar adenopathy), it is highly likely that a definitive diagnosis - probably of lymphoma - would have emerged, but at this stage he had no other symptoms. Our experience here helps emphasize the importance of tissue proof in simple cases - before and/or in case they become more complex. In more cryptic cases - and neurosarcoidosis can notoriously be difficult to differentiate from a wide range of other inflammatory and non-inflammatory conditions [1] - 'blind' biopsy of certain tissues such as conjunctiva and muscle can occasionally also yield diagnostic results (also reviewed in [1]).

In this patient, neurosarcoidosis was clinically an attractive explanation for his presenting problems: cough, hilar lymphadenopathy (confirmed by thoracic CT), development of a S1 radiculopathy, CSF abnormalities (raised protein level), bilateral lung hilar and lachrymal gland uptake on gallium scan, and erythema nodosum confirmed with skin biopsy, followed by the development of multiple cranial neuropathies, including seventh nerve palsy.

CSF abnormalities are seen in 80% of patients with neurosarcoidosis, but are non-specific; likewise, serum and CSF ACE (consistently normal in this patient) are also an insensitive test for sarcoidosis [1,4,5]. A brain MRI may show multiple non-specific white matter lesions, isointense on T1-weighted images, high signal intensity in T2-weighting, and with contrast enhancement of both the lesions and meninges.

The diagnosis of neurosarcoidosis requires a compatible clinical, laboratory and/or radiological picture of sarcoidosis and histological confirmation of non-caseating granulomas [6]. In 'probable' neurosarcoidosis (Table 1), as in our patient, aggressive or experimental therapy should be commenced only with very great caution and in particular in the case of tumour necrosis factor blockade, following the utmost efforts to exclude tuberculosis. The progressive neurological deterioration of our patient, with no opportunity for histological interrogation, was thought to warrant such a therapeutic approach. Unfortunately, this produced no beneficial results for the patient. Erythema nodosum, of course, is hardly specific to sarcoidosis, in particular, lymphoma is a recognized cause.

**Table 1:** Diagnostic criteria for neurosarcoidosis establish definite, probable and possible disease [6]

***Definite***	Clinical presentation compatible with neurosarcoidosis
	Exclusion of other possible causes
	Positive nervous system histology
***Probable***	*Clinical presentation compatible with neurosarcoidosis*
	Laboratory support of CNS inflammation*
	Exclusion of other possible causes
	Evidence of systemic sarcoidosis**
***Possible***	Clinical presentation compatible with neurosarcoidosis
	Exclusion of other possible causes

*High concentrations of CSF protein and high numbers of cells, the presence of oligoclonal bands, or MRI evidence compatible with neurosarcoidosis. **Positive histology or at least two indirect indicators from gallium scan, chest imaging, and serum angiotensin converting enzyme.

Neurolymphomatosis represents a unique subtype of extra-nodal lymphoma with localised invasion of cranial or peripheral nerves, plexuses or nerve roots. In the vast majority of reported patients, the disease is a large B-cell non-Hodgkin's lymphoma (NHL) [7]. Usually it develops in patients with widespread systemic NHL, but the nervous system may be the sole site, and patients often present without known lymphoma [8]. Despite the infrequent clinical presentation of neurological complications, autopsy studies indicate common involvement of the peripheral nervous system [9]. In our patient, serum LDH was consistently normal and CSF cytology was always unremarkable - but in fact, only a minority of cases has positive CSF cytology [10]. His low lymphocyte count may have represented a clue, but a bone marrow biopsy (even at the time his skin nodules had developed) was normal, as was FDG-PET scanning - again, not unusual in some instances of lymphoma [11].

Cyclophosphamide and steroid treatment in this patient could have suppressed his lymphoma and so delayed its diagnosis. Paradoxically, it is not impossible that he did have refractory sarcoidosis warranting aggressive chemotherapy, but then developed lymphoma, that is, he had two diseases, but we believe this to be unlikely and that he had lymphoma at the outset. Cyclophosphamide may contribute to tumourigenesis; alternatively infliximab, at least in patients with inflammatory bowel disease, has been implicated in the development of lymphoma [12] - though conversely, one epidemiologically robust study involving almost 20,000 patients with rheumatoid arthritis found that infliximab was not associated with a greater risk of lymphoma [13]. Indeed, infliximab is even considered a potential treatment in other haematological neoplastic disorders including myelodysplastic syndromes [14].

## Conclusions

Constant diagnostic vigilance and clinical surveillance is required in disorders such as neurosarcoidosis, and the importance of a tissue diagnosis - not obtained in this case until very late in the course and then excluding sarcoid - cannot be overemphasised.

## Abbreviations

CNS: central nervous system; CT: computed tomography; CSF: cerebrospinal fluid; EMG: electromyogram; sACE: serum angiotensin converting enzyme; MRI: magnetic resonance imaging; FDG-PET: f-positron emission tomography; NHL: non-Hodgkin's lymphoma.

## Consent

Written informed consent was obtained from the patient for publication of this case report and any accompanying images. A copy of the written consent is available for review by the Editor-in-Chief of this journal.

## Competing interests

The authors declare that they have no competing interests.

## Authors' contributions

ES analyzed and interpreted the patient data, and ES and NJS together prepared a draft and then finalised the manuscript. Both authors read and approved the final manuscript.
